# Fast Explainable Recommendation Model by Combining Fine-Grained Sentiment in Review Data

**DOI:** 10.1155/2022/4940401

**Published:** 2022-10-18

**Authors:** Ying Wang, Xin He, Hongji Wang, Yudong Sun, Xin Wang

**Affiliations:** ^1^College of Computer Science and Technology, Jilin University, Changchun 130012, China; ^2^Key Laboratory of Symbol Computation and Knowledge Engineering (Jilin University), Ministry of Education, Changchun 130012, China; ^3^College of Artificial Intelligence, Jilin University, Changchun 130012, China

## Abstract

With the rapid development of e-commerce, recommendation system has become one of the main tools that assists users in decision-making, enhances user's experience, and creates economic value. Since it is difficult to explain the implicit features generated by matrix factorization, explainable recommendation system has attracted more and more attention recently. In this paper, we propose an explainable fast recommendation model by combining fine-grained sentiment in review data (FSER, (Fast) Fine-grained Sentiment for Explainable Recommendation). We innovatively construct user-rating matrix, user-aspect sentiment matrix, and item aspect-descriptive word frequency matrix from the review-based data. And the three matrices are reconstructed by matrix factorization method. The reconstructed results of user-aspect sentiment matrix and item aspect-descriptive word frequency matrix can provide explanation for the final recommendation results. Experiments in the Yelp and Public Comment datasets demonstrate that, compared with several classical models, the proposed FSER model is in the optimal recommendation accuracy range and has lower sparseness and higher training efficiency than tensor models or neural network models; furthermore, it can generate explanatory texts and diagrams that have high interpretation quality.

## 1. Introduction

With the rapid development of Internet technology and industry, the amount of interactive information on the Internet has increased sharply, and the rate of interaction has also increased exponentially. Therefore, how to meet the personalized needs of different users is particularly important. For example, in online shopping websites, personalized recommendation system will directly guide users to participate in consumption, so it can create a win-win situation to meet users' needs and expand economic benefits [1].

Most of the existing recommendation systems [2–4] focus on optimizing targeted evaluation indicators, such as root mean square error, normalized discounted gain rate, and so on. These indicators focus on the error between the predicted user's rating of items and the real rating or the difference between the predicted user's preference for items and the real ranking. Obviously, the user's decision depends on a series of factors, and the quality of the index performance cannot specifically explain which factors affect the results. Although many algorithms have continuously improved the index performance [5–7], when the recommendation results are wrong, it is difficult to explain the reasons and convince users, resulting in a decline in user acceptance. Therefore, in recommendation algorithms, such as matrix factorization model, it is very important to mine the factors that affect user selection or can explain item quality, and the interpretability of recommendation has been paid more and more attention.

At present, the research on the interpretability of recommendation systems mainly considers two directions: user-oriented result interpretation and model-oriented principle interpretation. For example, ATM [8] models user preferences and item attributes on scoring and comment texts, obtains the importance of item attributes, integrates them into the item attribute oriented implicit feature model ALFM, and disperses users' preferences for items into users' preferences for item attributes as an explanation. EFM [9] aligns the decomposed implicit features with explicit item attributes so that the source of recommendation score can be interpreted as the result of the joint action of the advantages and disadvantages of item attributes and users' preferences for item attributes. In summary, an excellent recommendation algorithm should have a certain persuasive ability. Even if there is a deviation in the recommended item, it can still explain to the user why it is recommended and persuade the user to choose the item to a certain extent. In this regard, the work of this paper tends to explain the recommendation results to users; that is, it tends to be effective and persuasive in interpretability. The core is to tap the potential factors affecting the recommendation results, and the purpose is to generate the interpretation content that users can easily accept. More specially, when the model recommends an item to a user, it not only provides the user's possible rating, but also offers certain explanations from other aspects, such as the advantages and disadvantages of each attribute of the item in the review information. The key to solving the above problems lies in what and how to mine the factors affecting users' decision-making, and the recommendation accuracy should be guaranteed.

In this paper, we propose an interpretable fast recommendation model FSER ((Fast) Fine-Grained Sentiment for Explainable Recommendation), which integrates fine-grained emotion in comment text. We consider not only the user's rating, but also the influencing factors from the user's comment text, such as mining the item attributes and descriptors (adjectives, emotional words) in the user's comment text and modeling the user's fine-grained emotional factors of the item so as to enhance the persuasion of the recommendation system and the user's trust in the system. Mining fine-grained emotions from comments can not only help infer user preferences to improve recommendation accuracy, but also describe the advantages and disadvantages of each attribute of recommended items to generate intuitive explanations. Based on the recommendation method of matrix factorization, we construct three coupling matrices integrating fine-grained emotion for mutual constrained joint factorization so as to mine users' potential rating and fine-grained emotion. The main contributions of this paper are as follows:

1. We propose a user-item attribute emotion auxiliary score prediction method, which uses the shared implicit feature matrix to jointly decompose the user-item rating matrix and the user-item attribute emotion matrix. Experiments show that the recommendation accuracy is in the optimal range on Yelp and public comment datasets.
2. We propose to decompose the item attribute descriptor frequency matrix by using the shared item attribute implicit feature matrix to predict the word frequency score of each attribute descriptor. Combined with the fine-grained emotion of users on item attributes, the recommended interpretation text and illustration diagram are generated. Error analysis and cases verify the rationality and readability of the interpretation.
3. We propose a coupling interaction method to compress the quaternion interaction data into spliced binary metadata to construct a joint matrix factorization, that is, split (user-item-attribute-descriptor) into (user-item), (user-item attribute), and (item attribute-descriptor), so as to avoid the construction of fourth-order or third-order tensors and greatly reduce the data sparse value ratio and data storage. At the same time, when constructing the user-item attribute emotion matrix, the sparse value ratio of training data is reduced again by filtering irrelevant item attributes. In the time-consuming experimental analysis, the training efficiency of the model is greatly improved compared with the recommendation model using comment text data. The second section discusses the related work, and the third section is about problem statement. The fourth section develops the deep agent network GA, including model structure, training algorithm, and test algorithm. The fifth section gives the experimental results. Finally, we summarize the paper in the sixth section.

The rest of this paper is organized as follows: Section 2 discusses the related work.  Section 3 is about symbol definition and problem statement.  Section 4 develops the FSER model.  Section 5 gives the results of the experiments. Finally, we summarize the paper in Section 6.

## 2. Related Work

In this section, we briefly discuss the related work in interpretability of recommendation system, mainly about interpretable recommendation model based on matrix factorization and other types of interpretable recommendation models.

### 2.1. Interpretability of Recommendation System

With the in-depth study of the recommendation system, the effectiveness and accuracy of the recommendation results are gradually improved. However, the traditional recommendation system lacks a reasonable explanation of the recommendation results, which affects the user's acceptance of the recommendation results.

The research on the transparency of recommendation system [10] and the explanatory research on common collaborative filtering algorithms [11] have achieved some results. Early recommendation system interpretation relies on item content labels, which can be used not only for recommendation prediction, but also for user interpretation [12]. In 2014, Zhang et al. [9] formally defined interpretability: explain the working principle of the recommendation system or explain the results, enhance the transparency of the system, enable users to understand when the system makes mistakes (testability), help users make fast and high-quality decisions (effectiveness), and influence or persuade users to choose items (persuasiveness) and improve users' acceptance (satisfaction) of recommended items.

In recent years, the interpretability of recommendation system has been systematically classified and summarized [13]. According to different models, interpretable recommendation system can be divided into interpretable recommendation based on matrix factorization, interpretable recommendation based on topic, interpretable recommendation based on graph, and interpretable recommendation based on deep learning.

### 2.2. Interpretable Recommendation Model Based on Matrix Factorization

Matrix factorization algorithm and its variants have achieved great success in recommendation tasks, including classical implicit feature model (LFM) [14], singular value factorization model (SVD) [15, 16], nonnegative matrix factorization model (NMF) [17], probability matrix factorization model (PMF) [18, 19]. The recommendation prediction results of these models have high accuracy, but the factors decomposed by these models are an implicit feature, which is difficult to explain the reasons for recommendation to users, and users need to have a certain degree of trust in the recommendation system.

In order to make the personalized recommendation model intuitive and easy to understand, researchers pay more attention to the interpretable recommendation algorithm based on model or user, which can not only generate recommendation results, but also provide a variety of natural interpretation schemes. The explicit factor model (EFM) [9] aligns the decomposed implicit features with explicit item attributes so that the source of recommendation score can be interpreted as the result of the joint action of the advantages and disadvantages of item attributes and users' preferences for item attributes. Cheng et al. [8] applied the item attribute-oriented topic model (ATM) to model user preferences and item attributes on scoring and comment texts, obtained the importance of item attributes, integrated them into the item attribute-oriented implicit feature model (ALFM), and dispersed users' preferences for items into users' preferences for item attributes as an explanation. Wang et al. [20] proposed a multitask learning model, which uses the multitensor joint factorization method to predict the user's comments on each attribute of the item and uses the comments to give text interpretation.

### 2.3. Other Types of Interpretable Recommendation Models

Although the basic idea of the method used in this paper is different from the following methods, it is still a research worthy of reference in interpretative recommendation system. The topic based interpretable recommendation model mainly obtains the recommendation results and interpretation by analyzing and combining the content topic of the text. McAuley and Leskovec [21] proposed a method to align the implicit features obtained from recommendation factorization with the implicit topics of implicit Dirichlet distribution (LDA) to obtain recommendation interpretation. Lin Li et al. [22] proposed the DTMF + model, which establishes a positive mapping relationship between the potential topic vectors of user comment set and commodity comment set and the user potential factor vector and commodity potential factor vector of traditional matrix factorization and further guides the score prediction by adding potential topics. Tan et al. [23] put forward the concept of “recommendability” of goods. Based on the boosting method, the feature distribution representing the recommendability of goods is connected with the user's preference distribution in the same space. At the same time, user scores and comments are used for collaborative recommendation.

There are links between users, items, users and items, which is easy to build a graph structure. Particularly in social recommendation tasks, using graph structure to model users and items is more intuitive. He et al. [24] obtained by introducing the ternary relationship of tripartite graph modeling (user-item attribute) Top@K recommended model. Wang et al. [25] proposed the tree enhanced embedded model to obtain interpretable recommendations so as to combine the generalization ability of the embedded model with the interpretability of the tree model.

In recent years, deep learning and representation learning have attracted extensive attention, and an interpretable recommendation system based on deep learning technology has gradually emerged. Combined with CNN [26–32], RNN [33], memory network [34], and attention mechanism [35, 36], various recommended interpretation modes are generated. Beutel et al. [37] introduced the use of contextual information in recurrent neural networks (RNN) to improve the recommendation effect, conducted an experimental analysis of the classic feature extraction method, and applied RNN to enhance the efficiency of the recommendation algorithm. Costa et al. [38] designed a character-level recurrent neural network (RNN) model, which generates an item's review explanations using long short-term memory (LSTM). Chen et al. [39] propose visually explainable recommendation based on attentive neural networks to model the user attention on images, under the supervision of both implicit feedback and textual reviews.

## 3. Problem Statement

Let *U*={*u*_1_, *u*_2_,…, *u*_*m*_} and *V*={*v*_1_, *v*_2_,…, *v*_*n*_} be the sets of users and items, respectively, where *m* is the number of users and *n* is the number of items. We denote *R* ∈ *ℝ*_+_^*m*×*n*^ as a user-rating matrix for items, where *r*_*ij*_ = 1 if user *u*_*i*_ has interacted with item *v*_*j*_; otherwise, *r*_*ij*_=0. We use *S* ∈ *ℝ*_+_^*m*×*p*′^ to denote the user-item attribute emotion matrix of user's emotion towards each attribute of the item. And *O* ∈ *ℝ*_+_^*q*×*p*′^ denotes the descriptive word item attribute word frequency matrix when users comment on each item. The FSER model proposed in this paper aims to solve the problem of user recommendation in the real environment. When the user's scores and comments *D*={(*r*_*ij*_, *d*_*ij*_)} on some items are known, the possible scores of users on other untouched items are calculated, the items are recommended to users in turn according to the estimated scores, and a certain degree of interpretation information is obtained and fed back to users through this model. *D*={(*r*_*ij*_, *d*_*ij*_)} is the original dataset, and each data contains user-item score and comment text. *r*_*ij*_ represents the score of user *i* on item *j*. *d*_*ij*_ represents the comment text of user *i* on item *j*. The FSER model generation recommendation and interpretation process are defined as follows:

(1) $$\begin{array}{c} \Phi_{1}：R,S,O⟶U,A,A^{\prime },W⟶U,I,F,W\text{,}\\ \Phi_{2}：U,I,F,W⟶r_{ij},\text{Explanation}. \end{array}$$

Given *R*, *S*, and *O*, our goal is to predict users' unknown preferences towards items (i.e., the unobserved entries in *R*) by learning user and item representations and the user's preference for each attribute of the item is reconstructed to generate explanation text and explanation diagram.

## 4. Interpretable Recommendation Model with Fine-Grained Emotion

**Figure 1:**
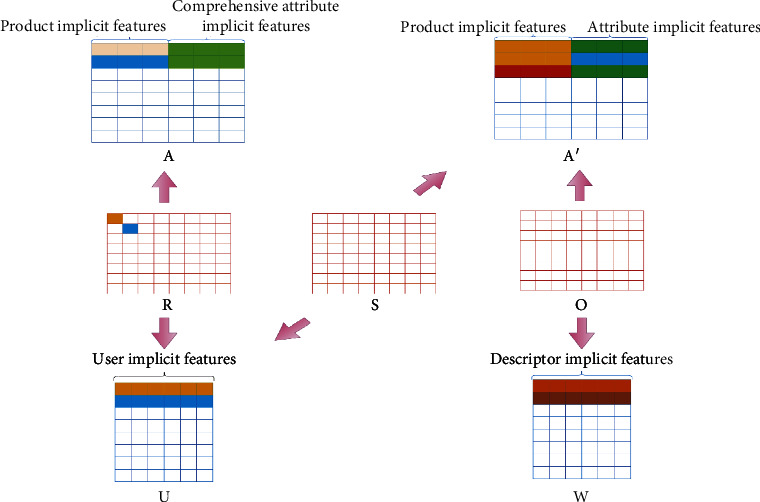
The joint factorization framework of each matrix in FSER.

The overall architecture of the proposed model is shown in Figure 1, which consists of two main components: (1) scoring prediction of joint user-item attribute emotion matrix and (2) recommendation interpretation generation of fused item attribute descriptors. The purpose of matrix factorization is to calculate the approximate dense matrix of the known sparse matrix to predict the missing content of the original matrix. This filling process is the factorization reconstruction method. The general matrix factorization recommendation algorithms are in lack of intuitive interpretation of the results. The factorization of the two matrices *S* and *O*, respectively, proposed in this paper can provide some explanation for the recommended results.

In Section 4.1, we describe in detail the process of constructing the user-item score matrix *R* ∈ *ℝ*_+_^*m*×*n*^ and the user-item attribute emotion matrix *S* ∈ *ℝ*_+_^*m*×*p*′^. In Section 4.2, the word frequency matrix *O* ∈ *ℝ*_+_^*q*×*p*′^ describing item attributes is constructed according to the description words in the comments. We use the same partial factorization factor as factorization *S* to carry out constrained joint factorization of matrix *O*. Finally, the overall optimization objectives and prediction formulas of the model are described in Section 4.3.

### 4.1. Scoring Prediction of Joint User-Item Attribute Emotion Matrix

The score-based matrix factorization needs to decompose the score matrix to obtain the implicit vector of users and items and predict the score. The formula for predicting the score between users and items represented by the implicit vector is defined as follows:

(2) $$\begin{array}{c} {\hat{r}}_{ui}=\overset{⟶}{u}\cdot \overset{⟶}{i.} \end{array}$$

#### 4.1.1. Constructing the User-Item Scoring Matrix *R*

**Figure 2:**
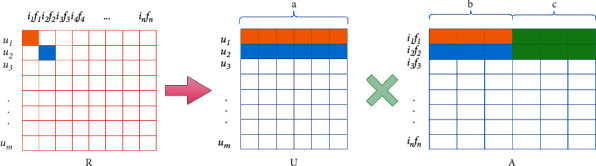
Implicit feature factorization of user-item scoring matrix *R*.

Similarly, we construct a sparse user-item score matrix *R* based on the user-item interaction data. *R*_*ij*_=*r*_*ij*_ represents the scoring value of the *i*th user on the*j*th item, and there is no scoring when *R*_*ij*_=0. The scoring matrix *R* is decomposed by the implicit factor factorization method, and the objective formula is as follows:

(3) $$\begin{array}{c} f_{1}=\underset{U,I,f}{argmin}\underset{\left(i,j\right)\in E}{\sum }{\left(R_{ij}-U_{i}A_{j}\right)}^{2}\text{,}\\ \begin{array}{cc} st. & A_{j}=\left[\begin{array}{cc} I_{j} & f_{j} \end{array}\right] \end{array},I\in R^{n*b},f\in R^{n*c},U\in R^{m*\left(b+c\right)}\text{,} \end{array}$$

where *U* is the feature matrix of users and *A* is composed of item implicit feature matrix *I* and comprehensive attribute implicit feature matrix *f*, as shown in Figure 2. Both *I* and *f* can represent the implicit features of the item itself, so the prediction score is

(4) $$\begin{array}{c} {\hat{r}}_{ij}=U_{i}^{\prime }\cdot I_{j}+U_{i}^{\prime \prime }\cdot f_{j}\text{.} \end{array}$$

#### 4.1.2. Constructing the User-Item Attribute Emotion Matrix *S* according to the Fine-Grained Emotion in the User Comment Text

When multiple (attribute descriptor emotion) triples (*k*, *w*, *s*)_*ij*_ are extracted from all comments of user *i*, user *i* often has more than one descriptor for attribute *k* of item *j* (*Q* represents the number of descriptors), *s*_*ijkw*_ represents the emotion for the *w*th descriptor of the attribute, and its value is +1 or −1 according to the emotional polarity of the descriptor. Define the overall emotion *s*_*ijk*_ of user *i* on attribute *k* of item *j*:

(5) $$\begin{array}{c} s_{ijk}=\overset{Q}{\underset{w=1}{\sum }}s_{ijkw},w=1,2,\ldots ,Q\text{,} \end{array}$$

where *s*_*ijk*_ ≥ 0 indicates that user *i* likes the attribute *k* of item*j*, and *s*_*ijk*_ ≥ 0 indicates that user *i* does not like the attribute *k* of item *j*.

However, there are two problems in the simple addition of emotions: (1) users' different preferences for items may be offset by the addition of positive and negative values. For example, for the screen attribute of mobile phone items, the user gives two descriptors *w*_1_ and *w*_2_, where if *w*_1_ is “bright color,” then *s*_1_=1. If *w*_2_ is “small,” then *s*_2_=−1. Add it to get the result of 0, which means that the user neither hates nor likes the item. However, the user's real score for the item may be 4 (likes), because compared with the screen size, the user cares more about whether the screen is bright, that is, |*s*_1_| > |*s*_2_|; the overall emotion should be greater than 0. (2) In the two comments with the same score and commented on the attributes of the same item, if the number of words describing the attributes is different, the way of adding emotional values will lead to a large gap between the two. Moreover, too many descriptors will lead to extremely large values. Too large gap and extreme value often affect the experimental effect and need smoothing.

It can be seen from problem (1) that users have different preferences for different attributes of items, and users' scores can reflect their preferences for various attributes in the items they actually pay attention to. On the contrary, the user's emotion for each item attribute is also the reference for predicting the user's final score, and the two are closely related. Therefore, constructing the user-item attribute emotion matrix and jointly training the user's emotion for item attributes and the user's score for each item can not only discover the user's preference for different item attributes, but also assist in score prediction, which can effectively solve problem (1).

When constructing the user-item attribute emotion matrix, it is considered that the sparse value mainly comes from the following two aspects: (1) a large number of users and items produce sparse values due to lack of interaction. It is determined by the actual data. It is the value to be predicted, and the sparsity cannot be reduced by deleting this value. (2) There are sparse values between items and attributes, because not every item can show all attributes, which is particularly serious in tensor model. It can be considered that, in a large number of comments on an item, if a certain attribute is not mentioned, the attribute may not exist in the item. For example, in the Yelp restaurant business dataset, beef and cheese exist in two different styles of restaurants: LFK (Lamesa Filipino Kitchen [‘Restaurants', ‘Filipino']) and CLKB (Cana Latin Kitchen and Bar ['Restaurants', ‘Latin American', ‘Tapas/Small Plates']). The user scores the attributes of the restaurant after eating, as shown in Table 1 (0 in the table indicates that the user does not score).

The nonnegative matrix factorization method is used to predict the user's preferences, as shown in Table 2. It can be seen that the error between the real score value and the predicted score value is less than 0.01, but the middle two columns in the table have no practical significance. It cannot be explained to the user that LFK beef is better than CLKB beef, or that the user likes items that do not exist in a restaurant.

**Figure 3:**
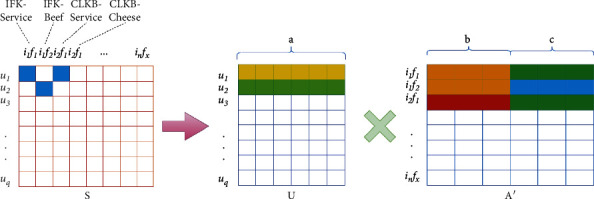
Implicit feature factorization of user-item attribute emotion matrix *S*.

Therefore, selecting meaningful (item attributes) according to a large number of user comments can reduce the nonexistent (item attributes) in the process of constructing the user-item attribute emotion matrix (matrix *S* in Figure 3), which not only alleviates the data sparsity, but also makes the recommendation results more practical.

For problem (2), the range of the median value in the emotion matrix is uneven, the emotion value can be smoothed by the sigmoid function, and its value can be mapped to the same interval as the score to reduce the influence of extreme value. The final user-item attribute emotion matrix is as follows:

(6) $$\begin{array}{c} S_{i\left(jk\right)}=\left\{\begin{array}{cc} 0\text{,} & \begin{array}{cc} if & \left(i,j,k\right)\notin E, \end{array}\\ 1+\frac{N-1}{1+\exp\;\left(-\tilde{s_{ijk}}\right)}\text{,} & others\text{,} \end{array}\right. \end{array}$$

where the value of *N* is generally the maximum value of the scoring range. For example, if the scoring range is 0–5, the value of *N* is 5. *S*_*i*(jk)_ represents the emotional value of user *i* on (item *j*-attribute *k*), which is obtained after smoothing. Each column in *S* represents the filtered (item attribute), with a total of *p*′ columns, as shown in the example given in the list in Figure 3, LFK beef and CLKB cheese, as well as the “service” attribute of both. And in most datasets, *p* ≪ *p*′ (*p* represents the number of attributes). The joint item attribute emotion matrix factorization shares the user implicit feature matrix *U* and the item implicit feature matrix *I*. The objective optimization formula is defined as follows:

(7) $$\begin{array}{c} f_{2}=\underset{U,I,F}{argmin}{\underset{\left(i,j,k\right)\in E}{\sum }\left(S_{i\left(jk\right)}-U_{i}A_{\left(jk\right)}^{\prime }\right)}^{2}\text{,}\\ \begin{array}{cc} st. & A_{\left(jk\right)}^{\prime }=\left[\begin{array}{cc} I_{j} & F_{k} \end{array}\right] \end{array}I\in R^{n*b},F\in R^{p*c},U\in R^{m*\left(b+c\right)}\text{,} \end{array}$$

where the generated implicit factor matrix *A*′ is composed of item implicit feature matrix *I* and attribute implicit feature matrix *F*. *A*_jk_′ represents a row in *A*′, which is the augmented splicing of item feature vector *I*_*j*_ and attribute feature vector *F*_*k*_. It is also of more practical significance to calculate the user's emotional prediction value of a certain attribute of the item.

By extracting the user's emotion for each attribute of the item, it can assist in the prediction. On the contrary, the user's score for each item can also help predict the user's emotion for each attribute of the item. The generated fine-grained user-item attribute emotion score can also be used as interpretation information to explain the recommendation results to users.

### 4.2. Recommendation Interpretation Generation of Fused Item Attribute Descriptors

User-item attribute emotion can not only assist the prediction of the recommendation system, but also predict the user's preference ranking of item attributes and generate a piece of explanatory information. For example, recommending item *j* for users is because users prefer the attributes *k*_1_, *k*_2_,…, *k*_*m*_ of item *j*. This paper further considers the descriptors of item attributes in the comments to find a more detailed explanation, that is, which words describe item attributes. The word frequency matrix *O* ∈ *ℝ*_+_^*q*×*p*′^ for describing item attributes is defined as follows:

(8) $$\begin{array}{c} O_{w\left(j,k\right)}=\left\{\begin{array}{cc} 0\text{,} & others\text{,}\\ 1+n\frac{\exp\;\left(\beta n_{w}\right)-\exp\;\left(-\beta n_{w}\right)}{\exp\;\left(\beta n_{w}\right)+\exp\;\left(-\beta n_{w}\right)}\text{,} & if\left(j,k,w,n_{w}\right)\in Set\text{,} \end{array}\right. \end{array}$$

where (*j*, *k*, *w*, *n*_*w*_) indicates that the number of times that the attribute *k* of item *j* in the comment data *d*_*ij*_ is described by the descriptor *w* is *n*_*w*_. Set is a set of (*j*, *k*, *w*, *n*_*w*_). *β* and *n* are all empirical parameters within the limited value range.

**Figure 4:**
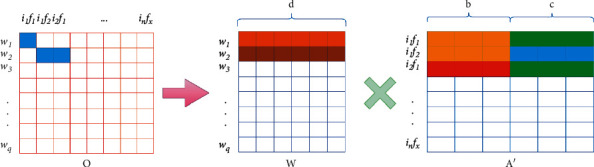
Implicit feature factorization of item attribute descriptor word frequency matrix *O*.

Each row in matrix *O* represents a word, and each column has the same meaning as each column in matrix *S*, representing the filtered (item attribute) combination. As shown in Figure 4, the implicit factorization of item attribute descriptor word frequency matrix *O* shares item implicit feature matrix *I* and attribute implicit feature matrix *F*. The objective optimization formula is as follows:

(9) $$\begin{array}{c} f_{3}=\underset{I,F,W}{argmin}{\underset{\left(w,j,k\right)\in E}{\sum }\left(O_{w\left(jk\right)}-W_{w}A_{\left(jk\right)}^{\prime }\right)}^{2}\text{,}\\ \begin{array}{cc} st. & A_{\left(jk\right)}^{\prime }=\left[\begin{array}{cc} I_{j} & F_{k} \end{array}\right] \end{array}I\in R^{n*b},F\in R^{p*c},W\in R^{q*\left(b+c\right)}\text{.} \end{array}$$

Here *W* is the implicit feature matrix of the descriptor. The joint word frequency matrix is decomposed into item and attribute implicit feature matrices, and the word frequency information of description words is integrated.

Finally, combined with the user's preference for each attribute of the item, the description and score of each attribute of the item are calculated. The experimental part verifies the rationality of the interpretation through the error analysis and example verification of fine-grained emotion prediction.

### 4.3. Objective Function and Prediction Formula

The overall framework of matrix combination is shown in Figure 1. The user score matrix *R* is decomposed to obtain the user feature matrix *U* and factor matrix *A*, and the item attribute descriptor word frequency matrix *O* is decomposed to obtain the descriptor feature matrix *W* and implicit factor matrix *A*'. The square of the *F*-norm is converted into a matrix description, which is equivalently integrated *f*_1_, *f*_2_, and *f*_3_, and regularized for each implicit feature matrix. Finally, the objective function of the FSER model is obtained as follows:

(10) $$\begin{array}{c} f_{FSER}=\underset{U,I,F,W,f^{*}}{argmin}\left\{{\left\|U^{T}A-R\right\|}_{F}^{2}+\lambda_{f}{\left\|U^{T}A^{\prime }-S\right\|}_{F}^{2}+\lambda_{w}{\left\|W^{T}A^{\prime }-O\right\|}_{F}^{2}+\lambda_{1}{\left\|U\right\|}_{F}^{2}+\lambda_{2}{\left\|I\right\|}_{F}^{2}+\lambda_{3}{\left\|F\right\|}_{F}^{2}+\lambda_{4}{\left\|W\right\|}_{F}^{2}\right\}\text{,}\\ st.A=\left[I\left[f^{*}\right]\right],A_{\left(jk\right)}^{\prime }=\left[I_{j}\;F_{k}\right],I\in R^{n\times b},F\in R^{p\times c},U\in R^{m\times \left(b+c\right)},\left[f^{*}\right]\in R^{n\times c},W\in R^{q\times \left(b+c\right)}\text{.} \end{array}$$

Compared with ${\hat{r}}_{ij}=U_{i}\cdot I_{j}$ in the traditional matrix factorization algorithm, the prediction score in FSER is shown in formula (4). The user's fine-grained emotion prediction value ${\hat{s}}_{ijk}$ for each attribute of the item is as follows:

(11) $$\begin{array}{c} {\hat{s}}_{ijk}=U_{i}\cdot A_{jk}^{\prime }=U_{i}^{\prime }\cdot I_{j}+U_{i}^{\prime \prime }\cdot F_{k}\text{.} \end{array}$$

Formula (4) combined with formula (11) shows the embodiment of fine-grained emotion in predicting user score, where *N*_*k*_ represents the number of attributes of item *j*.


(12) $$\begin{array}{c} {\hat{r}}_{ij}=U_{i}^{\prime \prime }\cdot {\overset{⟶}{f}}_{j}+\frac{1}{N_{k}}\overset{N_{k}}{\underset{k}{\sum }}\left({\hat{s}}_{ijk}-U_{i}^{\prime \prime }\cdot F_{k}\right)\text{.} \end{array}$$

It can be seen intuitively that the final prediction score is equal to the linear combination of the comprehensive attribute features of the user and the item plus the value related to the user's prediction score for each attribute of the item. Because ${\hat{s}}_{ijk}$ mainly comes from the factorization of fine-grained emotion *s*_*ijk*_ in the original data, the final prediction score is essentially constrained by the real emotion extracted from the original data; fine-grained emotion assisted prediction is used. Similarly, the score prediction of item attribute descriptor is shown in the following formula:

(13) $$\begin{array}{c} {\hat{o}}_{jkw}=W_{w}\cdot A_{jk}^{\prime }=W_{w}^{\prime }\cdot I_{j}+W_{w}^{\prime \prime }\cdot F_{k}\text{.} \end{array}$$

The item implicit matrix *I* and attribute implicit matrix *F* are obtained by using real score data and fine-grained emotion training, and the implicit matrix *W* of description words is trained at the same time. The construction of *O* matrix is similar to obtaining the word-of-mouth of item attributes in public so as to obtain the predicted word-of-mouth words and scores of item attributes as the explanation of recommendation to users.

When the data scale is large, the adaptive small batch gradient descent method is used to optimize the objective function, which can avoid falling into local optimization to a certain extent. The adaptive method can dynamically update the step size with the number of iterations. FSER model is shown in Algorithm 1.

## 5. Experiments

In this section, we perform comprehensive experiments to verify the effectiveness of our proposed method. We will first introduce the datasets, baselines, evaluation metrics, and experimental settings and then compare our proposed method with various baselines. Finally, we perform an example analysis of the interpretability of the model and conduct detailed ablation studies on the proposed method.

### 5.1. Datasets

In order to verify the effectiveness of the model in different scenarios, the experiment uses Yelp restaurant business dataset and public comment dataset. Yelp is a famous merchant review website in the United States. Users evaluate the star rating of merchants according to their own preferences and often leave comments. The merchants and contents of reviews are extensive. Public comment is a famous comment website in China, which involves many fields such as catering and accommodation, including users' scores and evaluation texts for merchants. Therefore, this paper uses English and Chinese data, respectively, and also contains recommended content in different fields. However, 54% of users in Yelp dataset and 49% of users in the public comment dataset only commented once. The item attributes and descriptors retained in the data processing process also have errors in natural language understanding. Therefore, when obtaining data, it is necessary to filter out representative data and filter unreasonable attributes and descriptors according to frequency. Finally, the extracted dataset is shown in Table 3.

### 5.2. Baselines

The baseline methods for comparison are funkSVD, PMF, baisSVD, EFM, SVD++, and MTER.

*FunkSVD*. The model is proposed by Funk and makes up for the defect of singular value factorization (SVD), which is not suitable for large-scale and sparse data matrix in recommendation system. Using the mean square error method to reconstruct the original matrix factorization into approximate matrix is the basis of most matrix factorization methods.

*PMF* [18]. Based on the regularized matrix factorization model (regularized MF), the model is further optimized by introducing a probability model. Assuming that the feature matrices of user *u* and item *i* obey Gaussian distribution, the feature matrices of *u* and *i* are obtained through the value of the scoring matrix, and then the unknown value in the scoring matrix is predicted by the feature matrix.

*BaisSVD*. On the basis of funkSVD, the model adds the scoring factors irrelevant to the item as the user bias term and participates in the scoring prediction with a certain weight.

*EFM* [9]. The model considers the relationship between each attribute of the recommended item and the user's score, uses joint matrix factorization to obtain the user's preference for each attribute of the item, and gives interpretable information for the score while giving the predicted score.

*SVD* ***++***. The model adds the potential preferences contained in the user score to the singular value matrix factorization algorithm in the form of implicit feedback; that is, the correction items related to all items evaluated by the user are added to the score prediction model.

*MTER* [20]. The model uses tensors instead of matrix factorization and uses the item attributes extracted in the comment text and user emotion description to construct the joint factorization of three tensors.

### 5.3. Evaluation Metrics

When the recommended result output is a real value, the commonly used recommended prediction evaluation indexes are root mean square error (RMSE) and mean absolute error (MAE). They respectively use root mean square and absolute error to evaluate the accuracy of prediction scores, which are defined as follows:

(14) $$\begin{array}{c} \text{RMSE}=\sqrt{\frac{\underset{i,j\in TestSet}{\sum }{\left(r_{ij}-{\hat{r}}_{ij}\right)}^{2}}{\left|TestSet\right|},}\\ \text{MAE}=\frac{\underset{i,j\in TestSet}{\sum }\left|r_{ij}-{\hat{r}}_{ij}\right|}{\left|TestSet\right|}\text{,} \end{array}$$

where |TestSet| is the number of test data samples and *r*_*ij*_ and $\hat{r_{ij}}$, respectively, represent the real score and predicted score of user *i* on item *j*.

### 5.4. Experiment Settings

**Figure 5:**
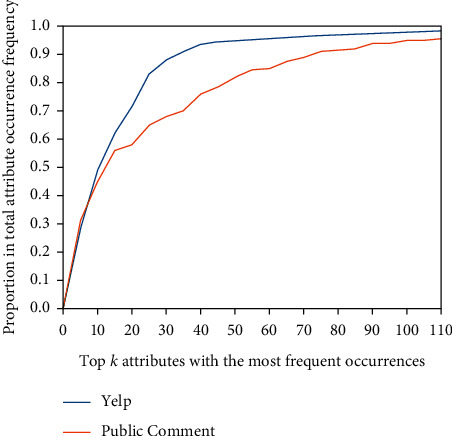
Proportion of main attributes in total attributes.

When extracting item attributes from the dataset, 1814 item attributes are extracted from the public comment dataset and 1065 item attributes are extracted from the Yelp dataset. Many attributes include attributes that are used less frequently and synonymous attributes, as well as a small number of unreasonable attributes caused by natural language processing errors. Before using attributes, synonymous attributes, such as power, battery, output, and battery life, can be classified as battery attributes. As shown in Figure 5, the occurrence frequency of a small number of attributes accounts for 80%–90% of the total occurrence frequency of attributes. Therefore, removing complex attributes during the experiment helps to reduce data noise and ensure that the main attributes cover more than 90% of the total occurrence frequency of attributes and increase frequency limiting filtering (e.g., attributes with filtering frequency less than 3000 in the public comment dataset). In the public comment and Yelp datasets, the main top 135 and top 104 attributes are retained, respectively. In addition, users, items, and descriptors are retained according to their frequency threshold in the dataset. The detailed filtering threshold and the average (reviewed) number of items and users are shown in Table 4.

The experimental parameters are set in a wide range, so the combination of random parameter search and set parameter search is used in the experimental process. The error parameters *λ*_*f*_ and *λ*_*w*_ are searched by random parameters. At the same time, in order to reduce the degree of experimental fitting and enhance the generalization performance of the model, make *λ*_1_=*λ*_2_=*λ*_3_=*λ*_4_=*λ*_reg_. In this experiment, *λ*_*f*_, *λ*_*w*_, and *λ*_reg_ are set to be 0.35, 0.8, and 5.4, respectively. The selection range of implicit factor dimension settings is still wide. In this paper, multiple groups of values within the range of 12–192 are fixed for search and comparison for qualitative rather than quantitative analysis.

### 5.5. Experimental Results

**Figure 6:**
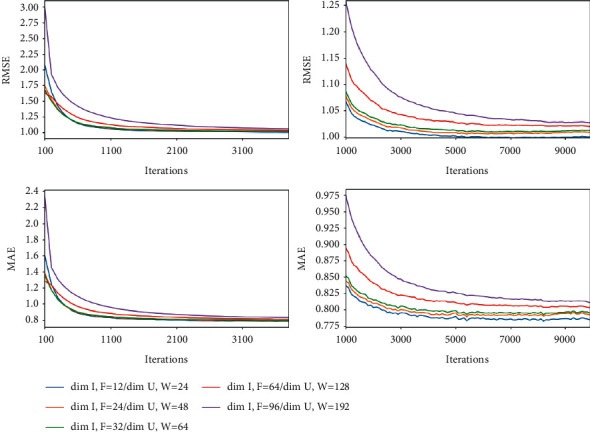
Influence of implicit feature dimension on experimental results.

The overall experimental results are shown in Figure 6. From Yelp data, it can be seen that when the implicit factor dimension setting is too large or too small, both RMSE and MAE are large at the beginning of training; the smaller dimension setting can obtain better results in the subsequent training. In order to make the explanation reasonable, the experiment makes nonnegative constraints on the obtained implicit vector in each batch of training.

The scale of the two datasets used in this paper is similar, so the public comment dataset is used to complete the training time comparison experiment under the same CPU and other hardware equipment.  Table 5 shows the time taken for the training error to reach their relative optimization in each model. As can be seen from the table, our proposed method FSER is stronger than SVD++, MTER, and EMF in terms of efficiency, while it is slightly weaker than PMF and funkSVD. This is probably because our proposed method incorporates two additional matrix factorizations. At the same time, Table 6 gives a comprehensive comparison of each model to illustrate the significance of the running time of each model.

According to the training time consumption, PMF, funkSVD, and FSER belong to the fast-training model, and SVD++ belongs to the acceptable training time-consuming range. However, SVD++ model adds correction items related to all items evaluated by users, and the size of dataset has a significant impact on its training speed. Therefore, the time consumption will be more serious when the data scale increases. The time consumption of MTER and EFM is relatively high, and it often takes dozens of hours of training to get better results. Among them, the tensor operation of MTER is relatively large, and the alternating iterative minimization training method adopted by EFM is more time-consuming when the data matrix is too large. It can be seen from Table 6 that although PMF and funkSVD are trained quickly, they only use user score prediction and do not contain any interpretation information. Although MTER and EFM have interpretation ability, their running speed is much slower than FSER. Therefore, the model proposed in this paper not only has the ability of interpretation, but also has the advantages of fast training and easy application, compared with other models.

### 5.6. Accuracy Analysis of Model Recommendation

**Figure 7:**
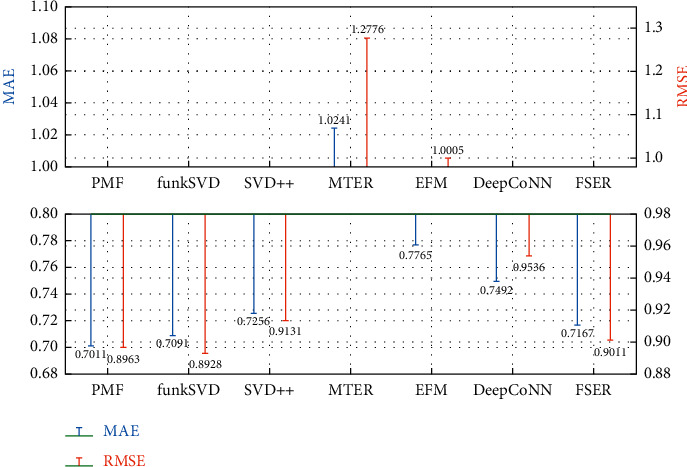
Prediction error result of public comment score.

**Figure 8:**
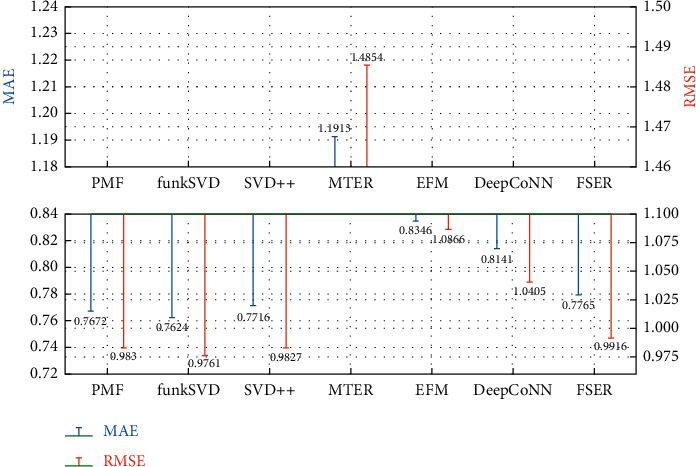
Prediction error result of Yelp score.

It can be seen from the experimental results in Figures 7 and 8 that the effect of using only the score prediction recommendation algorithms (PMF, funkSVD, and SDV++) is generally better. From the average number of user comments and the average number of item comments in Table 4, there are tens to hundreds of values in each row and column of the score matrix for factorization. Therefore, the data preprocessing makes the score matrix have good factorization prediction value. However, none of the above algorithms has the ability to interpret the recommended results. FSER model mines fine-grained emotion and description words in comment text to provide users with recommendation interpretation and also ensures that the recommendation accuracy is in the optimal range. It has a minimum difference of 0.0156 from the optimal MAE and 0.0083 from the RMSE in the public comment and Yelp datasets.

In addition, the results of fine-grained emotion prediction modeling of users by the model are shown in Table 7. The average absolute error and error between the predicted value and the real value of fine-grained emotion in Yelp dataset are 0.6004 and 0.8128, which means that when recommending items to users, the predicted score of users' preference for the attributes of the item is closer to the real situation, which further shows that FSER model has excellent interpretation ability.

### 5.7. Model Interpretive Analysis

Generally, interpretative evaluation schemes are divided into the online evaluation and offline evaluation, user research evaluation, and case study evaluation. Online evaluation mostly uses online user feedback, such as click, browse, questionnaire feedback, and so on. Offline evaluation mostly adopts random case analysis, comparative analysis, weakening the interpretation quality, and paying attention to the proportion of interpretable quantity in the results.

In order to analyze and verify the interpretation effect of FSER model, this paper adopts three common offline methods: interpretable quantitative analysis, comparative analysis, and case verification:

1. *Number of Interpretable Results*, Based on the reconstruction matrix, the FSER model can give the prediction score and interpretation of any user and item in the experimental data. EFM is the same as MTER. However, there is a lack of support for cold start data, and it is difficult to recommend and interpret untrained data.
2. *Comparative Analysis*. Recommendation explanation of EFM does not contain rich descriptors, but only the user's preference explanation for item attributes. Therefore, we compare the MTER model with better interpretation performance.  Table 8 shows the item attribute prediction emotion and description explanation given when the target user recommends an item. The interpretation descriptors generated by the MTER model are greatly affected by item attributes. For example, salsa (a kind of sauce) and other attributes in the table use almost the same recommendation words in different recommendation explanations. FSER model takes the combination of (items, attributes) as a set of features. When recommending for target users, the same attribute “beans” of different items are represented by different vectors, so a variety of explanatory words and weights are obtained.
3. *User-Recommended Examples*. The analysis of two groups of examples is given below.

The first group is the No. 6 target user recommending No. 5039 item (shown in Table 9), and the user's real score was 5. There were 168 comments on the item in the experimental data, and 20 were randomly selected for analysis, of which 17 had an evaluation score of 5, 2 had an evaluation score of 4, and 1 had an evaluation score of 3. Seven mentioned that they like “chocolate”; three mentioned “eating,” including “happy eating” and “eating heavy”; and one mentioned “Thai tea.” The target user thinks that “the shop is small but well organized.” Both “inside bright” and “inside small” appear in our explanation, which shows the user the attributes of the item. Secondly, the weight difference is large. It explains to the target user that “small” is not an important factor of the item. On the whole, the interpretation effect is obvious. However, the high-frequency core word “gelato” appears in various comments, which is “ice cream” in Italian. The word is not recognized by the Stanford thesaurus in the process of text extraction.

The second group is the No. 4333 target user recommending No. 985 item (shown in Table 10), and the user's real score is 4. In the experimental data, there are 26 item reviews, of which 7 have an evaluation score of 5, 6 have an evaluation score of 4, 11 have an evaluation score of 3, 1 has an evaluation score of 2, and 1 has an evaluation score of 1. The name of the restaurant “salad and go” is a restaurant mainly focusing on fast food and salad. Among them, 8 evaluations have commented on “food” many times, and its descriptive words are mainly “fast, health, fresh.” It can be seen from Table 9 that the interpretation given by FSER model is very close. In addition, there are 8 items describing “salad” as “seasonal” and 2 items describing it as “imaginary.” The descriptions of “salad” are relatively diverse, which is consistent with the low proportion of the scores of each descriptor in Table 9. In addition, the “options: blueberry” in the explanation, namely, “optional blueberry sauce,” is obviously very friendly and persuasive to users. The target user really commented on “Okay, focus on salads ... But I am here to announce their breakfast is fast and filling ... Also deeply healthy.” The  “food: fast, health” given priority by FSER obviously has good explanatory power.

To sum up, the FSER model generates various explanations and is close to different items and attributes. It has better interpretation ability than the existing matrix factorization interpretable recommendation model. The fine-grained emotion of the interpretation results provided to users can be effectively explained and can convince users to choose items to a certain extent, which is in line with the effectiveness and persuasion of the explanation we are concerned about.

### 5.8. Ablation Study

In order to better understand our proposed framework FSER, we investigate the impact of model components through ablation experiments. To further study the effect of the user-item attribute emotion matrix *S* and the word frequency matrix *O*, we set up a model without matrix *S* (w/*oS*) and a model without matrix *O* (w/*oO*), respectively, and conduct ablation experiments on the two datasets. The experimental results over the two datasets are given in Table 11. From the table, we can see that the model without matrix *S* and the model without matrix *O* all have a drop in recommendation performance compared with the proposed FSER. This phenomenon can indicate that the reconstruction of the matrix *S* and the matrix *O* not only provides an explanation for the recommended results, but also contributes to the model performance.

## 6. Conclusion

The speed of the recommended model and the amount of information it contains can determine the quality of the model. FSER model can not only effectively use the fine-grained emotion in the comment text to construct the joint factorization of user-item attribute emotion matrix, but also make the item attribute descriptors participate in the generation of interpretation text and interpretation diagram and retain the information contained in the comment text to a great extent. The experimental results show that the performance and recommendation quality of FSER model is better, the interpretation is more consistent with users, and the processing speed is faster in the multi-information fusion recommendation system.

Due to the complex relationship between information, the fusion of multiple information is often implicit. Artificial extraction and preprocessing may lead to the loss of information or the addition of irrelevant information. Therefore, we will further study how to convert high-quality multivariate data into training data in the real world. At the same time, we will explore the balance between the improvement of recommendation effect brought by the integration of multivariate information and the decline of training efficiency, as well as the balance between the improvement of interpretation ability and the increase in training cost.

## Figures and Tables

**Algorithm 1:**
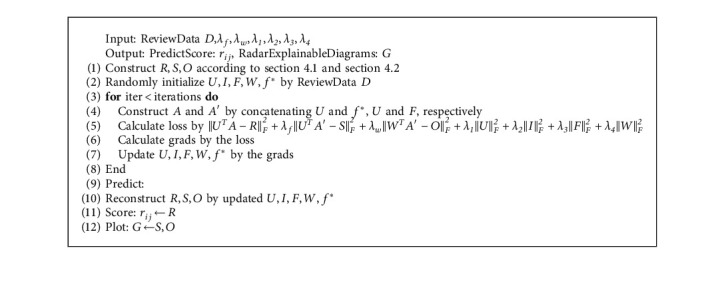
FSER.

**Table 1:** The user scores the attributes of the restaurant after eating.

Users	LFK beef	LFK cheese	CLKB beef	CLKB cheese
User 1	5	0	0	0
User 2	0	0	0	1

**Table 2:** Nonnegative matrix factorization to predict user score.

Users	LFK beef	LFK cheese	CLKB beef	CLKB cheese
User 1	4.9900625	1.53266015	0.89519114	2.82964816
User 2	1.68725349	0.59055061	0.29813681	0.99061711

**Table 3:** Datasets.

Dataset	Number of users	Number of items	Number of comments	Number of attributes	Descriptors
Public comment	11,753	17,241	1599,289	135	1,040
Yelp	10,719	10,410	468,630	104	1,019

**Table 4:** Data preprocessing.

Dataset	User filtering threshold	Average number of user comments	Item filter threshold	Average number of item reviews	Descriptor filter threshold	Proportion of descriptor extraction (%)
Public comment	>20	136.28	>35	92.76	>50	37.13
Yelp	>11	43.72	>25	45.02	>50	55.41

**Table 5:** Comparison of running time.

Model	Running time (s)
PMF	35.6
funkSVD	124.4
SVD++	1044.1
MTER	30804.8
EFM	>72 h
FSER	227.5

**Table 6:** Comprehensive comparison of models.

Model	Comment text	Fast training	Interpretative
PMF		**√**	
funkSVD		**√**	
SVD++			
MTER	**√**		**√**
EFM	**√**		**√**
FSER	**√**	**√**	**√**

**Table 7:** FSER fine-grained emotion prediction results.

Dataset	MAE	RMSE
Yelp	0.6004	0.8128
Public comment	0.7598	0.9577

**Table 8:** Comparison of the interpretation content generated by each model.

Model	User-item	Interpretation content
MTER	user0-item5858:	#salsa: good happy last noodle bland #staff …
	user10-item6454:	#salsa: good happy last noodle bland #store …

FSER	user6-item5336:	#beans: 4.55 red 1.47 fresh 0.74 # chocolate …
	user30-item7366:	#beans: 4.80 best 3.25 roasted 2.14 select 1.58 fresh 0.94 # pizza …

**Table 9:** FSER recommended explanation example (6-5039).

Item attributes	Score	Descriptors
Chocolate	4.35	Dark 5.82 strawberry 4.75 covered 2.3 white 1.4
Inside	4.33	Bright 6.77 eating 5.03 small 0.06
Eating	4.30	Heavenly 6.27 happy 2.40 inside 1.81
Salmon	4.29	Smoked 2.69
Tea	4.29	Thai 3.35

Real comment		6RIj37bU37ZTX8yKVFYugg

**Table 10:** FSER recommended explanation example (4333–985).

Item attributes	Score	Descriptors
Food	5.22	Fast 1.462 healthy 1.3235 good 1.1308
Cheese	5.03	Candied 2.166 blue 1.5796
Salad	4.67	Seasonal 1.2514 fantastic 1.1901 healthy 1.0454 good 0.9333 great 0.8816
Drink	4.65	Unique 1.1359
Options	4.62	Blueberry 1.4028 unique 1.1674

Real comment		Ek2AlcFM5zX-hFjFS3I9EA

**Table 11:** Results of ablation experiments (without *S* and *O*).

Dataset	Metric	FSER		W/*oS*	W/*oO*
Public	MAE	0.7167	0.7283		0.7203
Comment	RMSE	0.9011	0.9254		0.9108

Yelp	MAE	0.7795	0.7926		0.7851
	RMSE	0.9916	1.1123		0.1069

## Data Availability

Data are available at https://github.com/syd951186545/FSER/tree/master/Data.
